# Complex Roles of Solution Chemistry on Graphene Oxide Coagulation onto Titanium Dioxide: Batch Experiments, Spectroscopy Analysis and Theoretical Calculation

**DOI:** 10.1038/srep39625

**Published:** 2017-01-03

**Authors:** Shujun Yu, Xiangxue Wang, Rui Zhang, Tongtong Yang, Yuejie Ai, Tao Wen, Wei Huang, Tasawar Hayat, Ahmed Alsaedi, Xiangke Wang

**Affiliations:** 1 School of Environment and Chemical Engineering, North China Electric Power University, Beijing, 102206, P.R. China; 2 University of Science and Technology of China, Jinzhai road 96, Hefei, 230000, P.R. China; 3 NAAM Research Group, Faculty of Science, King Abdulaziz University, Jeddah 21589, Saudi Arabia

## Abstract

Although graphene oxide (GO) has been used in multidisciplinary areas due to its excellent physicochemical properties, its environmental behavior and fate are still largely unclear. In this study, batch experiments, spectroscopy analysis and theoretical calculations were addressed to promote a more comprehensive understanding toward the coagulation behavior of GO onto TiO_2_ under various environmental conditions (pH, co-existing ions, temperature, etc.). The results indicated that neutral pH was beneficial to the removal of GO due to the electrostatic interaction. The presence of cations accelerated GO coagulation significantly owing to the influence of electrical double layer compression. On the contrary, the presence of anions improved the stability of GO primarily because of electrostatic repulsion and steric hindrance. Results of XRD, FTIR and XPS analysis indicated that the coagulation of GO on TiO_2_ was mainly dominated by electrostatic interactions and hydrogen bonds, which were further evidenced by DFT calculations. The high binding energy further indicated the stability of GO + TiO_2_ system, suggesting that TiO_2_ can be used as an effective coagulant for the efficient elimination and coagulation of GO from aqueous solutions. These findings might likely lead to a better understanding of the migration and transformation of carbon nanomaterials in the natural environment.

With the fast development of technology and dramatic enhancement in the production of manufactured nanomaterials, the carbon-based nanomaterials (e.g., carbon nanotubes, carbon nanofibers, carbon dots and graphene) have obtained intriguing attention for their commercial, electronic, and medical applications^1^,^2^,^3^,^4^. Graphene has been extensively termed as the “most exciting nanomaterial” due to its remarkable properties (e.g., mechanical strength, electrical and thermal conductivities etc.) ref. 5. Graphene oxide (GO), one of the most important graphene derivatives, has abundant epoxy and hydroxyl functional groups at its basal plane, as well as carbonyl and carboxyl groups situated at the edges^6^. The existences of these functional groups make GO highly hydrophilic, which permits GO easily swell and disperse in water^7^. The unusual structure and superior physicochemical properties of GO maintain its great promises in multidisciplinary areas, such as water treatment^8^, nanosensors^9^, supercapacitors^10^ and nanomedicine^11^. However, on the other hand, the released GO is unavoidable to be toxic pollutant during manufacturing, developing and disposing processes^12^.

The GO in the natural environment may directly or indirectly affect human health and ecosystems. Recent studies have demonstrated the toxicity of GO to different kinds of organisms. Ren *et al*.^13^ found that GO at ultra-trace concentration (0.01 μg/L) in water could trigger Parkinson’s disease-like symptoms and metabolic disturbance in zebrafish larvae. Tu *et al*.^14^ demonstrated that GO could induce cell damage in *E. coli* by extracting phospholipid molecules from the outer cell membrane. Liao *et al*.^15^ reported that GO showed the greater hemolytic activity of hemoglobin from suspended red blood cells as compared to the aggregated GO. Hence, the understanding of the physical and chemical behavior of GO in the natural environment is significantly to evaluate its toxicity to human beings. From the literature survey, the efficient way to lower the GO concentration in water is the coagulation of GO, which forms large agglomerates that can be easily separated by aggregation, filtration or centrifugation^12^,^15^,^16^. Thereby, the coagulation kinetics and stability of GO have been extensively investigated in the literatures^4^,^17^,^18^,^19^, and the results showed that the stability of GO in aqueous solution was strongly influenced by ionic strength and pH values. In our group, the aggregation behaviors of GO on Al_2_O_3_ and layered double hydroxides (LDHs) indicated that the stability of GO was strongly dependent on solution conditions and the coagulation was mainly dominated by the electrostatic interaction and hydrogen bonds^12^,^16^,^20^. Similarly, some works also reported that the solution conditions affected the fate and transport of GO in porous media^4^,^21^,^22^,^23^. However, to the best of our knowledge, the coagulation behavior of GO on titanium dioxide (TiO_2_) under complicated aqueous environmental conditions have not been reported yet, especially the study from theoretical calculations.

As a multifunctional material, TiO_2_ has already been proved to have outstanding performances in photocatalytic^24^,^25^,^26^ and energy areas^27^,^28^,^29^. It has also been widely served as an environmental friendly model mineral for the removal of environmental contaminants due to its well-known properties such as high chemical and structural stability, abundant sources, low solubility at the whole pH range and close to neutral pH for point of zero charge^30^. Particularly, the composites of TiO_2_ and carbon-based nanomaterials are currently recognized as highly effective adsorbent and catalyst in the purification of wastewater^24^,^31^,^32^,^33^. Moon *et al*.^34^ prepared the rGO-TiO_2_ composites based on pH-induced aggregation for the efficient photocatalytic oxidation of arsenite. Wang *et al*.^35^ observed the accelerated sedimentation of TiO_2_-GO and attributed it to the aggregation of TiO_2_-GO resulted from the electrostatic attraction between TiO_2_ and GO. These studies indicated that TiO_2_ may be suitable materials for GO coagulation from solution to solid particles. However, systematic studies on the interaction mechanism between TiO_2_ and GO over a broad range of conditions have not been reported heretofore. In addition, spectroscopic evidence and theoretical calculations are crucial to understand the interaction mechanism at molecular level, which is significant to evaluate the migration behavior of GO in natural environment. Such knowledge is significant to better assessment the environmental behavior of toxic GO nanomaterials and to provide basis for further investigation of carbon-based nanomaterials.

The objectives of the current study are: (1) to study the influence of pH, co-existing ions, contact time, temperature and TiO_2_ contents on GO coagulation onto TiO_2_; (2) to characterize the microscopic surface changes before and after GO deposition on TiO_2_ using scanning electron microscopy (SEM), X-ray diffraction (XRD), Fourier transformed infrared spectroscopy (FTIR) and X-ray photoelectron spectroscopy (XPS); (3) to further confirm the interaction mechanisms of GO with TiO_2_ by density functional theory (DFT) calculations. It is a highlight to understand the coagulation behavior of GO onto TiO_2_ through the investigation of macroscopic experiments, spectroscopy analysis and theoretical calculations. Furthermore, the coagulation behavior offers new insight into the interaction of GO with TiO_2_, which can further understand the behavior of carbon-based materials at solid/water interfaces in natural environment.

## Results and Discussion

### Influence of solution pH

**Figure 1:**
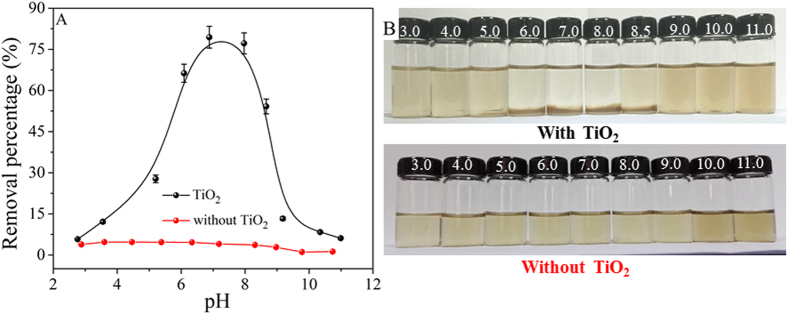
(**A**) Removal percentages of GO as a function of pH in the absence and presence of TiO_2_. (**B**) Photograph illustration of the influence of pH on GO coagulation after 24 h. C_(GO)initial_ = 25 mg/L, m/V = 0.1 g/L, T = 25 °C.

The coagulation behaviors of GO in the presence and absence of TiO_2_ at different pH values were shown in Fig. 1. The concentration of Ti ions in the solution was analyzed using an inductively coupled plasma mission spectroscopy (ICPE-9000, Shimadzu). It was found that Ti was not detected at 3.0 < pH < 11.0, indicating that TiO_2_ owned a good chemical and structural stability. In the absence of TiO_2_, it was clear that the deposition of GO was negligible (Fig. 1A), suggesting that GO was very stable in aquatic solution, which was also evidenced from the visually express in Fig. 1B. The results demonstrated that the GO suspension was very stable in a wide pH range, which was consistent with the literature^17^. However, after adding TiO_2_ to GO suspension, one can see that the deposition of GO increased sharply from 5% to 80% as the pH increased from 3.0 to 7.0, and then decreased greatly from 80% to 6% in the pH range of 7.0–11.0. To provide more intuitive evidence about the coagulation process of GO, the reaction photographs of GO with TiO_2_ as a function of pH were also presented in Fig. 1B. At low and high pH values, the suspension was homogeneous with a light claybank color, suggesting the good stability of GO suspension. Apparent precipitation was formed at neutral pH, suggesting the strongly deposition of GO onto TiO_2_ at neutral pH values. Similarly, the coagulation of GO onto Al_2_O_3_ and LDHs was also dependent on pH due to the electrostatic interaction, hydrogen bond and Lewis acid-base interaction^12^,^16^,^20^. According to the zeta potential analysis (Figure S2), the point of zero charge (pH_PZC_ = 6.0) of TiO_2_ indicated that the surface of TiO_2_ was mainly positively charged at pH < 6.0, whereas the GO was negatively charged at the whole pH. Therefore, the enhanced coagulation of GO onto TiO_2_ at pH 2.0–6.0 can be attributed to electrostatic attraction between negatively charged GO and positively charged TiO_2_. The high level deposition of GO at pH 6.0–8.0 was ascribed to the strong chemical interaction and electrostatic interaction^16^,^20^. At pH > 8.0, deprotonation of carboxylic groups was found to play a vital effect on the stability of GO^16^,^18^,^36^. Furthermore, the concentration of OH^−^ in aqueous solution increased with pH increasing, which can compete with GO for interaction with the functional groups on TiO_2_. In consequence, the negatively charged GO was difficult to be attached to the negatively charged TiO_2_ at high pH values due to the strong electrostatic repulsion, which resulted in the stable dispersion of GO in aqueous solutions.

### Influence of cations

**Figure 2:**
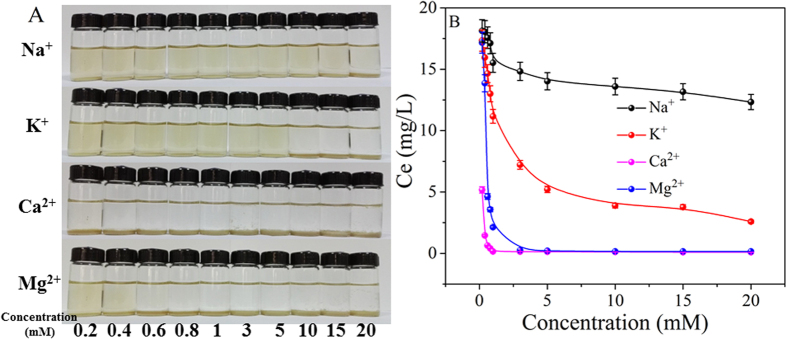
(**A**) Photograph illustration of the influence of cations on GO coagulation onto TiO_2_ after 24 h. (**B**) Concentrations of the residual GO in the supernatant as a function of the cation concentrations. C_(GO)initial_ = 25 mg/L, m/V = 0.1 g/L, pH = 5.0 ± 0.1, T = 25 °C.

The common environmental cations (Na^+^, K^+^, Ca^2+^ and Mg^2+^) were introduced to investigate the coagulation behavior of GO onto TiO_2_. Figure 2A visually expressed the influence of cation types and concentrations on the coagulation of GO. From Fig. 2A, one can see that GO was homogeneously dispersed in low cation concentration and visible precipitation was formed as the cation concentration increased to a critical value (e.g. 20 mM Na^+^, 10 mM K^+^, 0.2 mM Ca^2+^ and 0.6 mM Mg^2+^). In order to supply more quantitative knowledge about the deposition behaviors of GO, the equilibrium concentration (*C*_e_) of GO in supernatant at different electrolyte concentrations (NaCl, KCl, CaCl_2_, and MgCl_2_) were shown in Fig. 2B. GO was stable at relatively low concentrations (e.g. 0.01 to 1.0 mM) for monovalent cations and the difference between Na^+^ and K^+^ was negligible. Above 1.0 mM, the obvious differences among Na^+^ and K^+^ were observed, and the removal percentage of GO increased notably with increasing Na^+^ or K^+^ concentration. The maximum removal percentage of 51% in the presence of Na^+^ and that of 90% in the presence of K^+^ were found. Compared to monovalent cations, the significant deposition of GO was observed in the presence of Ca^2+^ and Mg^2+^ ions, i.e., as low as 0.2 mM for Ca^2+^ and 0.4 mM for Mg^2+^. The destabilization of GO in the presence of different kinds of cations were in the sequence of Ca^2+^ > Mg^2+^ >> K^+^ > Na^+^. However, Schulze-Hardy rule showed the similar deposition effect of the same valent cations^18^, which was a little different to the results of this work. This may be attributed to the different interaction properties of cations with GO, and thereby resulted in the more complicated coagulation properties of GO on TiO_2_ particles. Wu *et al*.^18^ also showed that the aggregating/destabilizing capacity of divalent cations were higher than monovalent cations and the reason was that divalent cations can interact with the functional groups on GO surfaces, particularly at the edges. The concentrations of Na^+^ and K^+^ in most natural water environment are less than 10 mM, therefore GO will be very stable and have strong migration potential in aqueous solution if no other cations are present in the natural water^37^. While, Ca^2+^ and Mg^2+^ concentrations in natural aquatic environments often over 0.1 mM, the common environmental divalent cations are more aggressive in GO destabilization, and then results in the deposition of GO in natural water conditions^18^,^22^. This behavior may be due to the strong binding capacity of divalent cations to functional groups of GO^38^.

### Influence of anions

**Figure 3:**
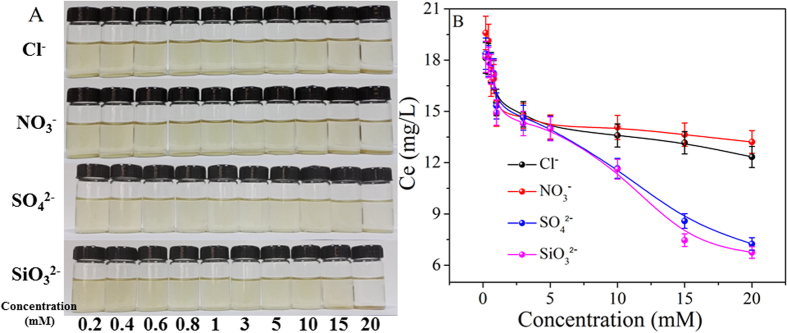
(**A**) Photograph illustration of the influence of anions on GO coagulation onto TiO_2_ after 24 h. (**B**) Concentrations of the residual GO in the supernatant as a function of the anion concentrations. C_(GO)initial_ = 25 mg/L, m/V = 0.1 g/L, pH = 5.0 ± 0.1, T = 25 °C.

To further understand the interaction mechanisms of GO with TiO_2_ under different solution chemistry conditions, the coagulation process under different concentrations of co-existing anions were shown in Fig. 3. Figure 3A revealed a direct residual GO concentrations in the supernatant after reaction with TiO_2_ for 24 h in the presence of different anions and concentrations. It is clear to see that the anions (NO_3_^−^, Cl^−^, SO_4_^2−^ and SiO_3_^2−^) had little influence on GO deposition, the minor difference was attributed to their negligible difference on Na loadings. The anion-π interactions between GO and anions occurred as GO can act as an electron acceptor for anion sorption^39^. Shi *et al*.^40^ demonstrated that the anion-π bond was unpredictable strong using a computational method on the basis of density functional theory. Furthermore, Hao *et al*.^41^ reported that 7,7,8,8-tetracyanoquinodimethane had strong π-π stacking interactions with graphene which can effectively prevent the inter- or intra-π-π stacking of graphene, leading to the dispersion of functionalized graphene. Therefore, the negligible effect of anions on GO deposition can be attributed to the strong anion-π interactions which prevented the agglomeration of GO and leading to the excellent water dispersibility. Besides, the anions may be adsorbed on the positively charged surface of TiO_2_ through electrostatic attraction, and the surface-adsorbed anions blocked parts of active sites on TiO_2_ surface and thus prevented the coagulation of GO due to the steric effects and enhanced electrostatic repulsion.

In the presence of anions, the coagulation of GO can be attributed to a balance between the anions (NO_3_^−^, Cl^−^, SO_4_^2−^ and SiO_3_^2−^) and Na^+^. The presence of anions resulted in the more negative surface charge of GO and stabilized GO in solution, while the increase of Na^+^ concentration leaded to the deposition of GO due to electrostatic attraction^12^. With the increase of electrolyte concentrations, the increased Na^+^ concentration had more pronounced influence on the stability of GO than the increased anions (NO_3_^−^, Cl^−^, SO_4_^2−^ and SiO_3_^2−^). From Fig. 3B, one can see that the final values of *C*_e_ slowly decreased as the electrolyte concentration increased. Compared with NaCl and NaNO_3_, each mole of Na_2_SO_4_ and Na_2_SiO_3_ includes two moles of Na^+^, therefore, the coagulation of GO onto TiO_2_ in the presence of different anions are in the sequence of SO_4_^2−^ ≈ SiO_3_^2−^ > Cl^−^ ≈ NO_3_^−^.

### GO coagulation

**Figure 4:**
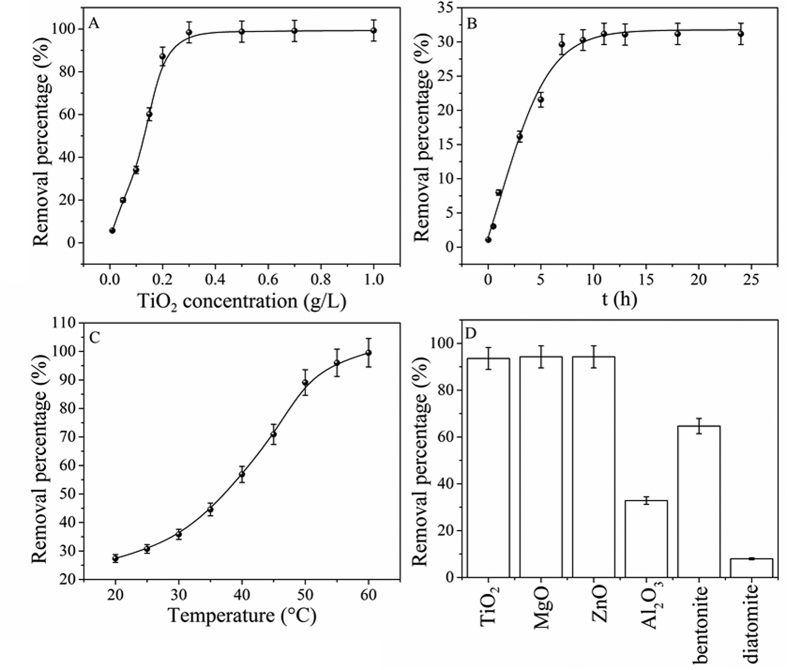
Coagulation of GO onto TiO_2_. (**A**) Effect of TiO_2_ concentrations, T = 25 °C; (**B**) Effect of contact time, m/V = 0.1 g/L and T = 25 °C; (**C**) Effect of temperature, m/V = 0.1 g/L; and (**D**) Comparison of GO coagulation capacities on different materials, m/V = 1 g/L, T = 25 °C; C_(GO)initial_ = 25 mg/L and pH = 5.0 ± 0.1.

Solid content is an important parameter in the removal of pollutants due to the limited amounts of functional groups and binding sites available for pollutant uptake^16^,^42^. From Fig. 4A, one can see that the removal percentage of GO increased from 5% to 99% as the TiO_2_ content increased from 0.01 to 0.3 g/L. The removal percentage of GO increased sharply at C[TiO_2_] < 0.3 g/L, and then a flat curve increased slowly at C[TiO_2_] > 0.3 g/L. The effective precipitation and polymerization between TiO_2_ and GO led to the quickly increase of GO deposition with TiO_2_ content increasing at C[TiO_2_] < 0.3 g/L^43^. At C[TiO_2_] > 0.3 g/L, most of GO was attached to the surface of TiO_2_, thereby the removal of GO from solution to solid particles increased slowly with TiO_2_ concentration increasing.

Figure 4B showed the removal of GO from aqueous solutions by TiO_2_ at pH 5.0 ± 0.1 as a function of contact time. The deposition of GO increased quickly with the increase of contact time and reached saturation after 10 hours of contact time. At initial contact time, large amount of functional groups on the surfaces of TiO_2_ were available for the binding of GO, which resulted in the quick uptake of GO to TiO_2_ surfaces. More aggregation sites and functional groups on TiO_2_ were available for the coagulation of GO at the initial contact time, and the deposition of GO on TiO_2_ was easily and quickly. With increasing contact time, the available sites were occupied by GO and thereby the uptake of GO became slow. The fast aggregation velocity suggested that strong chemisorption devoted to the deposition of GO onto TiO_2_, which was significant for the application of TiO_2_ to remove GO from aqueous solutions in natural environment.

To further gain insight into the coagulation behavior of GO in aqueous solution, the removal percentage of GO at different temperatures were shown in Fig. 4C. It was clear that the temperature played an important role on the deposition of GO and distinct precipitate was formed at T > 50 °C (Figure S3). According to Fig. 4C, the removal percentage of GO on TiO_2_ increased from 25% to 99% with the temperature increased from 20 to 60 °C, indicating that higher temperature was benefical for the aggregation of GO.

The removal percentage of GO on different coagulants were shown in Fig. 4D. One can see that TiO_2_ had similar deposition capacity with ZnO and MgO, however it was clearly to see that there were some suspended solids on the bottom of MgO and on the top of ZnO (Figure S4). The results indicated that the deposition of GO to TiO_2_ was more stable than to ZnO and MgO. In addition, it was interesting to notice that TiO_2_ has higher deposition capacity as compared with natural clay materials (e.g. bentonite and diatomite). On the basis of aforementioned analysis, it is clearly that TiO_2_ can be potentially used as a cost-effective coagulant for the efficient elimination of GO from aqueous solutions, which could efficiently decrease the potential toxicity of GO in the natural environment.

### Spectroscopic analysis

**Figure 5:**
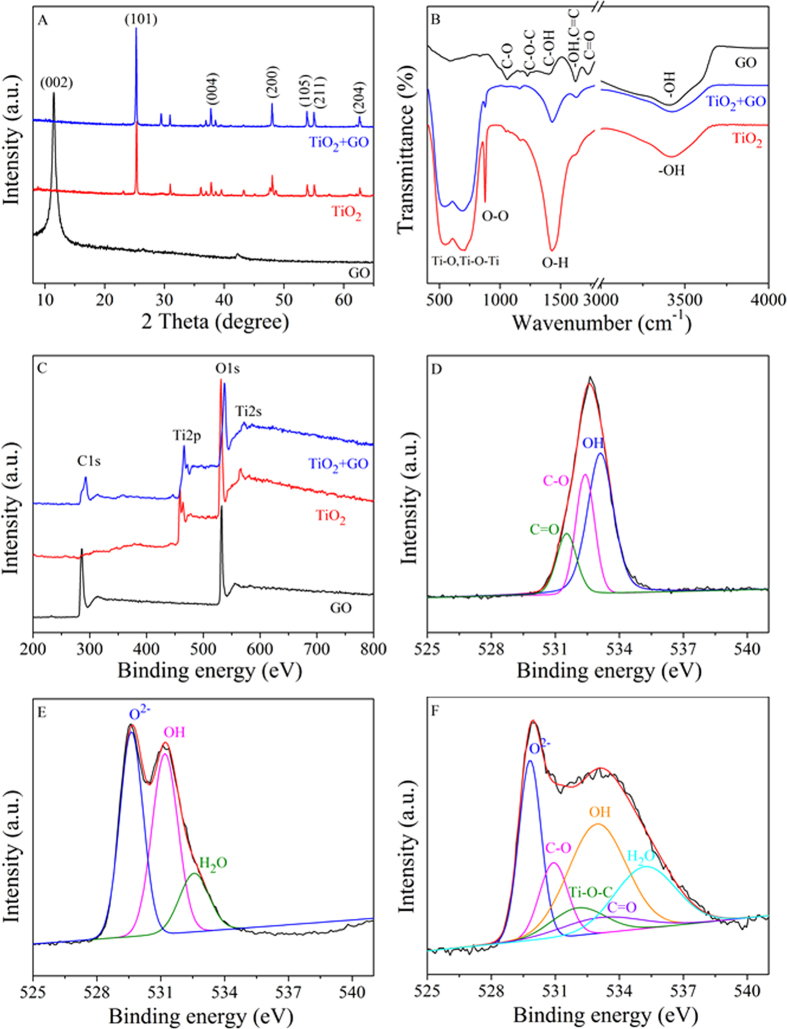
The characterization of GO, TiO_2_ and TiO_2_ + GO. (**A**) XRD patterns; (**B**) FTIR spectra; (**C**) XPS survey spectra. O 1s XPS spectra of GO (**D**), TiO_2_ (**E**) and TiO_2_ + GO (**F**).

To help deduce the interaction mechanism of GO with TiO_2_, the XRD, FTIR and XPS analysis of TiO_2_, GO and the TiO_2_ after GO coagulation (TiO_2_ + GO) were collected and compared in detail. The XRD patterns were shown in Fig. 5A. In the XRD pattern of TiO_2_, the peaks at 25.3°, 37.8°, 48.0°, 53.9°, 55.1° and 62.7° were indexed to the typical representations of the anatase phase of (101), (004), (200), (105), (211) and (204) reflections (JCPDS card No. 21-1272), respectively^24^. The diffraction peaks of TiO_2_ shifted slightly after GO coagulation, indicating that small crystal sized TiO_2_ was formed, similar results were also obtained in hybrid TiO_2_@rGO^28^. However, the representative (002) reflection at 2θ = 11.4° of GO was imperceptible, implying a decreased layer-stacking regularity and a highly disordered overlay of individual GO nanosheets were formed in the TiO_2_ architecture after GO aggregation^31^,^44^. Furthermore, from the SEM images (Figure S5), it was obvious that the surface of TiO_2_ was composed of stacked GO nanosheets, revealing that GO had been deposited on the surface of TiO_2_.

As can be seen from the FTIR spectra in Fig. 5B, the GO showed various adsorption bands for water -OH stretching (3400 cm^−1^), carboxylates or ketones C = O stretching (1730 cm^−1^), water -OH bending and C = C stretching (1627 cm^−1^), alcoholic C-OH bending (1400 cm^−1^), epoxide C-O-C (1230 cm^−1^) and C-O stretching (1057 cm^−1^)^26^,^45^. The fundamental vibrations of TiO_2_ appeared at 400–900 cm^−1^ which were ascribed to the stretching vibrations of Ti-O and Ti-O-Ti bonds, the peak at 3420 cm^−1^ was due to the stretching of the hydroxyl group^46^,^47^. The sharp peak at 1432 cm^−1^ was a characteristic band of hydroxyl group deformation vibration^45^. The band at 877 cm^−1^ may be due to O-O vibration, indicating the existence of peroxide bond at TiO_2_ surface. For TiO_2_ + GO sample, the absence of C = O, C-O and C-O-C bands indicated that strong chemical bonds were formed between TiO_2_ and GO^34^. Compared with pure TiO_2_, the intensities of O-H (1432 cm^−1^) and O-O (877 cm^−1^) vibration bands decreased after GO aggregation, suggesting the formation of hydrogen bond (O-H…O) between GO and TiO_2_^25^,^48^. Furthermore, the appearance of graphene skeleton peak at 1627 cm^−1^ (C = C stretching) and the slightly shift of Ti-O-Ti bond (400–900 cm^−1^) demonstrated the formation of Ti-O-C bonds. This behavior had also been reported for the anchoring of TiO_2_ nanoparticles on graphene nanosheets because of the strong chemically bond between GO and TiO_2_^49^. The FTIR analysis provided indirect evidence that GO was deposited on the surface of TiO_2_ through chemisorption and hydrogen bond.

The interaction mechanisms of GO with TiO_2_ were further investigated by XPS. The chemical properties of different elements in TiO_2_, GO and TiO_2_ + GO were shown in Fig. 5C–F. The survey spectra (Fig. 5C) showed clearly the existence of C, O in GO, Ti, O in TiO_2_ and C, Ti, O in TiO_2_ + GO. In addition, the binding energy values of Ti2p and O1s of TiO_2_ + GO remarkably shifted to higher wavelength as compared with TiO_2_, which was presumably due to strong chemical interaction between TiO_2_ and GO^16^. Similarly, Razzaq *et al*.^50^ observed that the Ti2p and O1s peaks of rGO + TiO_2_ shifted to higher binding energies as compared to pure TiO_2_, and they proposed that the drainage of electrons from Ti to rGO and the formation of bonds between TiO_2_ with rGO. The high resolution of O 1s spectra were shown in Fig. 5D–F. The O1s spectrum of GO (Fig. 5D) can be divided into three components located at 533.1, 532.4 and 531.5 eV which corresponded to the -OH, C-O (epoxy and hydroxyl) and C = O (carbonyl and carboxyl) groups, respectively^51^. The O 1s spectrum of TiO_2_ (Fig. 5E) was assigned to lattice oxygen O^2−^ (529.6 eV), terminal -OH (532.2 eV) and adsorbed H_2_O (532.6 eV), respectively^36^. As shown in Fig. 5F, the terminal -OH and lattice oxygen O^2−^ shifted to higher binding energy compared to the O1s spectrum of TiO_2_ before GO aggregation, and the -OH and O^2−^ appeared at the binding energies of 533.0 and 529.8 eV, respectively, suggesting that hydrogen bond was formed between TiO_2_ and oxygen functional groups on GO^25^. At the same time, the new peaks of C-O and C = O appeared in the O 1s spectrum of TiO_2_ + GO, which suggested that GO had been deposited on the surface of TiO_2_^16^. Interestingly, the new peak appeared at 532.2 eV can be attributed to Ti-O-C bond formed by the TiO_2_ and carboxyl group of GO, confirming the presence of GO in TiO_2_ + GO^50^. A prominent decrease in the peak intensities of C-O and C = O were observed in case of TiO_2_ + GO as compared to GO, further suggesting the interaction between TiO_2_ and surface functional groups of GO.

### DFT calculation

**Figure 6:**
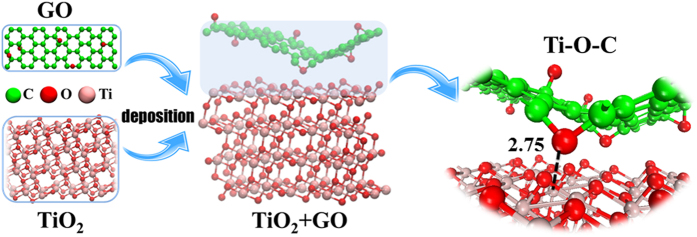
The optimized structures for the system of TiO_2_ + GO.

The interaction mechanism of GO onto TiO_2_ was further evidenced by the DFT calculations. The Vienna ab initio simulation package (VASP) (version 5.3.5) was implemented to perform the geometric optimization and static total energy calculations for the coagulation models^52^,^53^,^54^. Computational details were summarized in SI. The optimized structure of GO deposition on TiO_2_ was shown in Fig. 6. Owing to the oxygen-containing functional groups of GO plane, the minimum Ti(TiO_2_)-O(GO) distance was 2.75 Å. The binding energy (*E*_b_) (Table S1) was calculated as the following: *E*_b_ = *E*_GO_ + 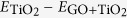, where *E*_GO_, 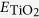 and 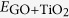 represent energies of the coagulation GO, TiO_2_ and the hybrid TiO_2_ + GO system, respectively. A high positive *E*_b_ between GO and TiO_2_ (5.79 eV) suggested that the TiO_2_ + GO system was stable and the chemisorption was probably the main coagulation mechanism of GO to TiO_2_, complementing to the physisorption behavior^16^, which indicated that strong interaction existed between GO and TiO_2_, and TiO_2_ was an effective coagulant for the elimination of GO from natural environment.

**Figure 7:**
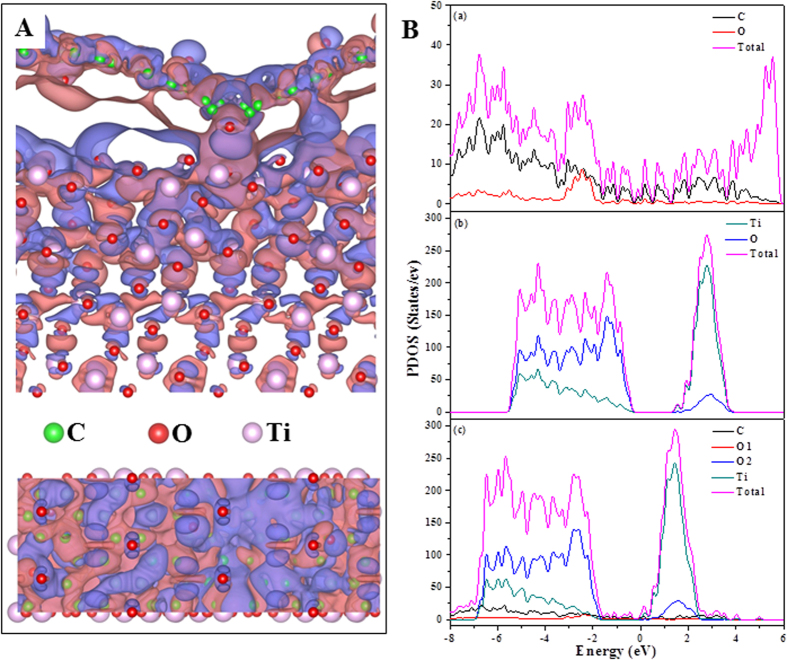
(**A**) The distribution of differential charge density between TiO_2_ and GO with the isovalue of 10^−5^ e Å^−3^. Purple and blue represent positive and negative charges, respectively. Above is the side view and below is the top view. (**B**) Partial density of states (PDOS) of (a) GO, (b) TiO_2_ and (c) TiO_2_ + GO system. O1 is the oxygen of GO and O2 represents the oxygen in TiO_2_.

The calculated charge density distribution and the projected density of states (PDOS) of the hybrid TiO_2_ + GO system were shown in Fig. 7. As shown in Fig. 7A, the three dimensional charge density difference plot with an isosurface value of 10^−5^ e Å^3^ was obtained by subtracting the calculated electronic charges of the individual GO and TiO_2_ (101) from that of TiO_2_ + GO. The purple and blue bubbles represented positive and negative charges, respectively. It can be clearly seen that the electrostatic interaction was formed between GO and TiO_2_. With further Bader charge analysis^55^, we discovered that there was an average charge transfer of around 0.06 e from GO to TiO_2_ (101) facets. Other significant evidence of the notable charge transference between GO and the TiO_2_ (101) facets was the comparison between the PDOS plots for GO, TiO_2_ and TiO_2_ + GO. As shown in Fig. 7B(c), from −7 to −2 eV range, there was an obvious hybridization between C, O and Ti orbitals. Because of the interaction and redistribution, the peaks of O1 and C atoms in TiO_2_ + GO system almost disappeared when compared with GO. Meanwhile, the peaks of O2 and Ti atoms in TiO_2_ + GO system shifted to lower energy levels compared to pure TiO_2_. The theoretical calculation suggested the presence of strong interactions between TiO_2_ and GO. Comprehensive consideration of the results of XRD, FTIR, XPS and theoretical calculations, it proved that chemical bonds, hydrogen bonds and electrostatic interactions dominated the coagulation of GO on the surface of TiO_2_ from aqueous solutions.

In conclusion, this paper is the first study to investigate the coagulation behavior of GO onto TiO_2_ under different environmental solution conditions. Electrostatic interaction is found to play a key role in GO removal by TiO_2_ when pH changes. Divalent cations are more effective than monovalent cations in aggregating/destabilizing GO suspensions through the interaction with oxygen-containing functional groups on GO surfaces. GO remains highly stable under different anions due to electrostatic repulsion and steric hindrance. The spectroscopy analysis and DFT calculations further evidence the electrostatic interactions and hydrogen bonds between GO and TiO_2_. The abovementioned insights into the coagulation of GO onto TiO_2_ under different solution chemistry conditions are crucial toward understanding the long-term transport and fate of GO.

## Methods and Materials

### Materials and characterization

GO was synthesized from flake graphite (48 μm, 99.95% purity) by using the modified Hummers’ method^56^. More detailed procedures about the preparation of GO were supplied in Supporting Information (SI). Milli-Q water was used in all experiments.

The TiO_2_ before and after GO coagulation were characterized by the scanning electron microscopic (SEM), X-ray diffraction (XRD), Fourier transform infrared spectroscopy (FTIR) and X-ray photoelectron spectroscopy (XPS). More detailed characterization processes were described in SI.

### GO Coagulation Experiments

The coagulation experiments were accomplished in a series of 20 mL vials equipped with Teflon-lined screw caps at 25 ± 1 °C by using batch technique. A certain amount of TiO_2_ (0.1 g/L), GO stock suspension (25 mg/L) and the background electrolytes (NaCl, KCl, MgCl_2_, CaCl_2_, NaNO_3_, Na_2_SiO_3_ or Na_2_SO_4_) (0–20 mM) were added to the vials to obtain the required concentrations of different components, and the samples were left undisturbed on a flat surface for 24 h to allow for the complete settlement of TiO_2_ and the large GO aggregates. The desired pH was adjusted by adding negligible volumes of 0.01 or 0.1 M HCl and NaOH. The detail experimental process was described in SI. The concentration of GO was analyzed by ultraviolet-visible spectrophotometer (UV-2550, PerkinElmer) at wavelength of 230 nm (Figure S1). All experimental data were obtained by the average values of triple parallel samples and the error bars were within ±5%.

## Additional Information

**How to cite this article**: Yu, S. *et al*. Complex Roles of Solution Chemistry on Graphene Oxide Coagulation onto Titanium Dioxide: Batch Experiments, Spectroscopy Analysis and Theoretical Calculation. *Sci. Rep.* **7**, 39625; doi: 10.1038/srep39625 (2017).

**Publisher's note:** Springer Nature remains neutral with regard to jurisdictional claims in published maps and institutional affiliations.

## Supplementary Material

Supporting Information
