# Supplementary material for: Complex Roles of Solution Chemistry on Graphene Oxide Coagulation onto Titanium Dioxide: Batch Experiments, Spectroscopy Analysis and Theoretical Calculation
Source: Sci Rep. 2017 Jan 3;7:39625. doi: 10.1038/srep39625 (PMC5206720; doi:10.1038/srep39625)
Supplement: Supporting Information [file srep39625-s1.doc]

**Supporting Information on**

**Complex Roles of Solution Chemistry on Graphene Oxide Coagulation onto Titanium Dioxide: Batch Experiments, Spectroscopy Analysis and Theoretical Calculation**

Shujun Yu^1,2^, Xiangxue Wang^1,2^, Rui Zhang^1,2^, TongtongYang^2^, Yuejie Ai^1*^, Tao Wen^1^, Wei Huang^1^, Tasawar Hayat^3^, Ahmed Alsaedi^3^, Xiangke Wang^1,3*^

1. School of Environment and Chemical Engineering, North China Electric Power University, Beijing, 102206, P.R. China

2. University of Science and Technology of China, Jinzhai road 96, Hefei, 230000, P.R. China

3. NAAM Research Group, Faculty of Science, King Abdulaziz University, Jeddah 21589, Saudi Arabia

1. The preparation of GO

The GO was synthesized by chemical oxidation of expanded graphite using modified Hummers method^1^. Typically, 2.0 g of flake graphite (48 µm, 99.95% purity) and 1.5 g NaNO_3_ (as co-solvent) were added into a 250 mL round bottom flask, and then 150 mL concentrated H_2_SO_4_ was added under stirring and ice-water bath conditions. Then 9.0 g of the oxidizing agent (KMnO_4_) was slowly added into the suspension more than 2 h. The suspension was continually stirred for 5 d at room temperature. Then 280 mL of 5 wt % H_2_SO_4_ was added and the temperature was kept at 98 °C for 2 h. 12 mL of H_2_O_2_ (30 wt %) was added in the suspension when the temperature was reduced to 60 °C. After reactions, the mixture was centrifuged and washed with 10 % H_2_SO_4_ solution to remove residual metal ions. The precipitate was then washed with distilled water and centrifuged repeatedly until the solution became neutral. The GOs were obtained by centrifuging at 18000 rpm for 60 min after ultrasonic treatment at 400 W for 30 min.

The chemicals, i.e., titanium dioxide (TiO_2_), calcium chloride (CaCl_2_), magnesium chloride hexahydrate (MgCl_2_·6H_2_O), potassium chloride (KCl), sodium chloride (NaCl), sodium nitrate (NaNO_3_), sodium sulfate (Na_2_SO_4_), sodium metasilicate nonahydrate (Na_2_SiO_3_·9H_2_O), sodium hydroxide (NaOH) and hydrochloric acid (HCl) were purchased from Sinopharm Chemical Reagent Co., Ltd. All chemicals used in the experiments were purchased in analytic purity and used without any further puriﬁcation.

2. Characterization

The SEM image was recorded on a scanning electron microscope (S-4300). The XRD pattern was recorded on a MAC Science Co. M18XHF diffractometer using Cu Kα radiation (λ = 0.154 nm). FTIR spectrum was performed by a Bruker Tensor-27 spectrophotometer in the range of 4000-400 cm^-1^ using the KBr disc technique. The XPS spectrum was recorded on an ESCALAB 250Xi spectrometer from Thermo Scientific Ltd with monochromatized Al Kα source operated at 200 W.

3. Batch Coagulation Experiments

The coagulation experiments are accomplished in a series of 20 mL vials equipped with Teﬂon-lined screw caps at 25 ± 1 °C by using batch technique. A certain amount of TiO_2_ (0.1 g/L), GO solution (25 mg/L) and/or the background electrolytes (NaCl, KCl, MgCl_2_, CaCl_2_, NaNO_3_, Na_2_SiO_3_ or Na_2_SO_4_) (0-20 mmol/L) added to the vials and the samples are settled down for 24 h. The desired pH of the suspensions in each vial is adjusted in the range of 3.0-11.0 by adding negligible volumes of 0.01 or 0.1 mol/L HCl and NaOH solutions. The UV-vis absorption spectroscopy results for GO are presented in Figure S1. The optimum wavelength of GO was determined to be 230 nm (Figure S1(A)), and an R^2^ > 0.9998 for the calibration curve of GO at 230 nm suggested that the GO absorbance results can be directly correlated to their concentrations (Figure S1(B)). Each experimental data is obtained by the average values of triple parallel samples (the resulting error bars (within ± 5%) are provided). The amount of GO coagulated on TiO_2_ was calculated from the difference between the initial concentration (C_0_) and the equilibrium one (C_e_). Removal percentage (R) was calculated as R (%) = (C_0_ - C_e_) / C_0_ × 100%.

4. Computational details

The graphene model is built by the unit cell parameters of graphite: *a=b=*2.460 Å, *c=*6.800 Å; *α=β=*90°, *γ=*120°. The unit cell parameters of the anatase TiO_2_ is *a=b=*3.776 Å, *c=*9.486 Å; *α=β=γ=*90°. A four-layer slab was used with the atoms in the bottom two layers were fixed to their bulk positions with a vacuum layer of 2.5 nm. In order to model the interaction between the GO and the TiO_2_ surface, a rectangular super cell of graphene (a’ = 5b - 5a, b’ = 3a + 3b) was built to fit the 2× 2 supercell of TiO_2_ (101) surface, the final lattice parameters of the graphene supercell are *a’*=21.304 Å, *b’*=7.38 Å. The GO are constructed randomly by oxygen atoms at the bridge site with a C:O ration of 10:1. The optimized structure of GO has been shown in Figure S6.

Plane-wave-based DFT calculations on slab models are carried out by projector augmented wave (PAW) method with the generalized gradient approximation (GGA) and Perdew-Burke-Ernzerhof (PBE) functions^2,3^. The cutoff energy was used at 400 eV in present calculations. The self-consistent field iterations were considered to be converged when the change of total energy is smaller than 10^-5^ eV. The force convergence criterion was set to 0.02 eV/Å. For geometry optimizations, the Brillouin zone was sampled with a Monkhorst-Pack mesh of 3 × 3 × 1 k-points^4^.


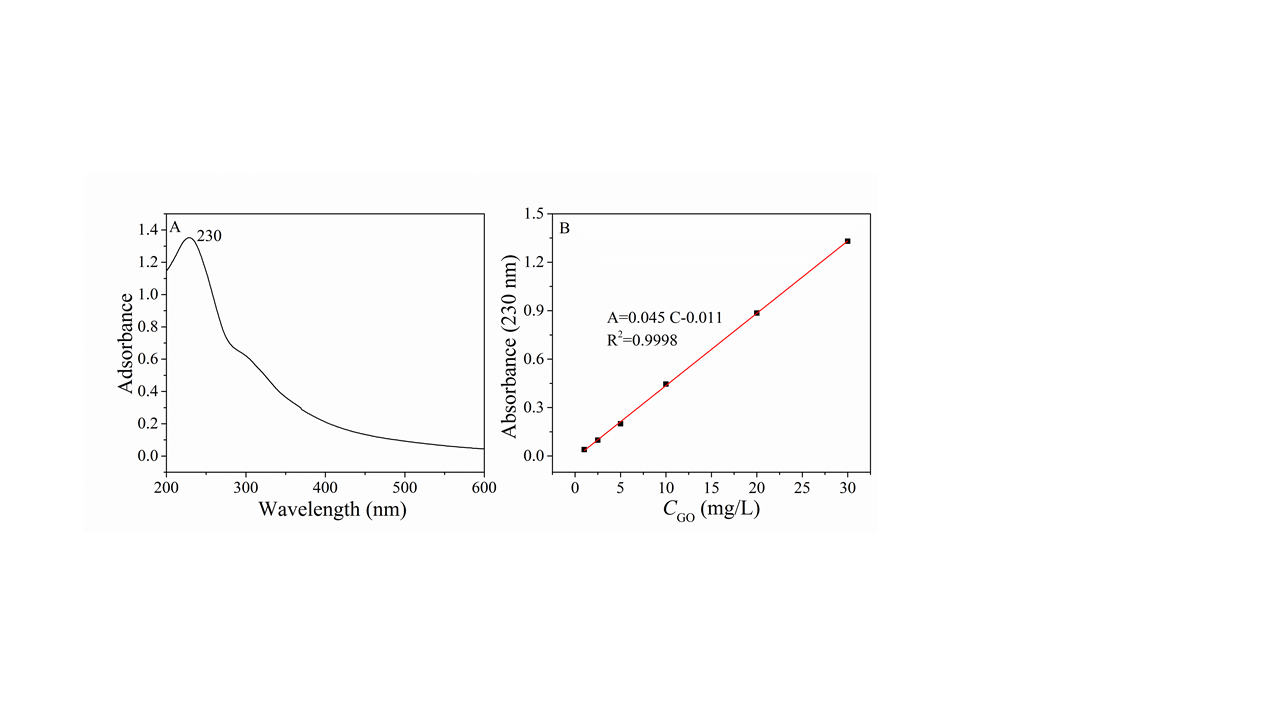


Figure S1. (A) The UV-vis absorption spectroscopy of GO. (B) Absorbance as a function of GO concentration at the wavelength of 230 nm.


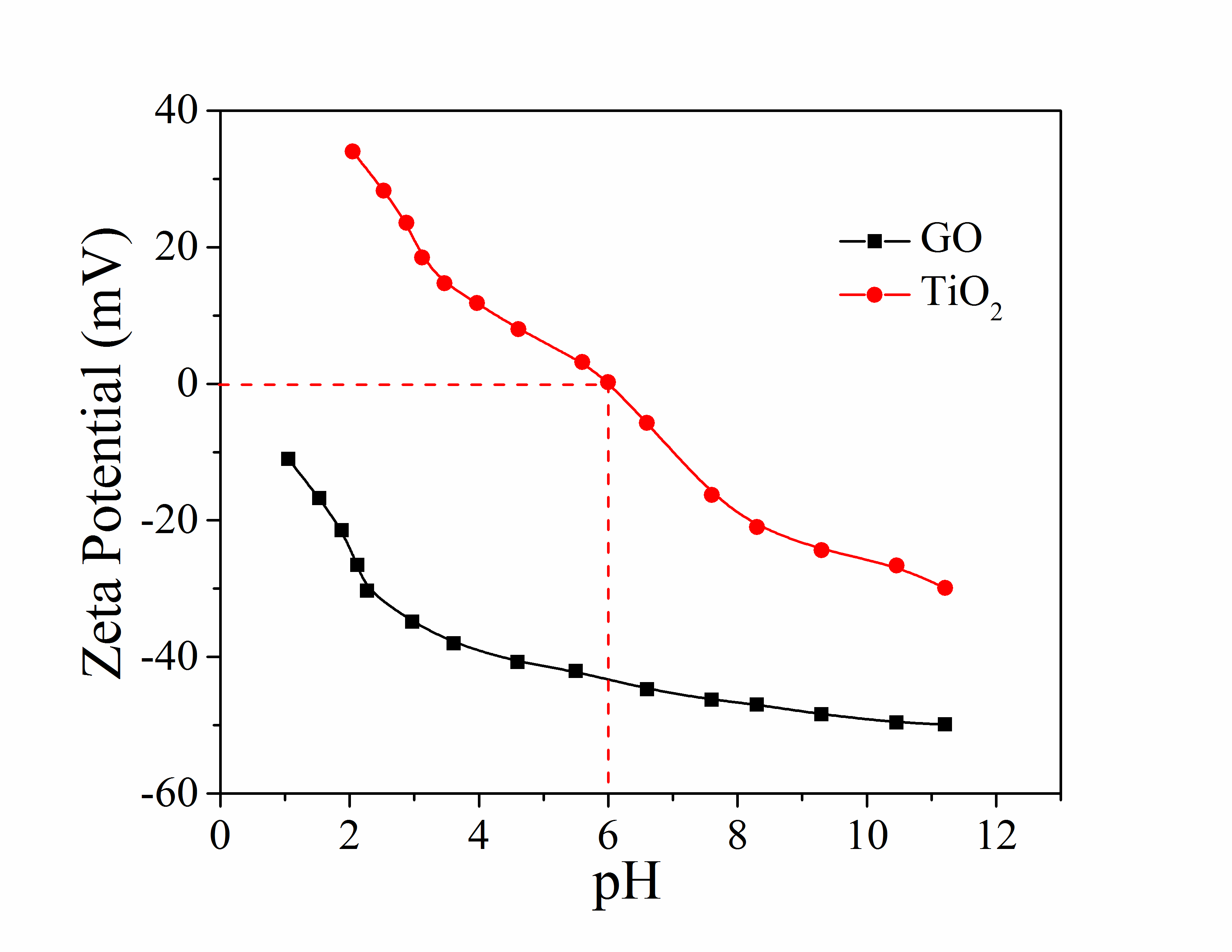


Figure S2. Zeta potential of GO and TiO_2_.


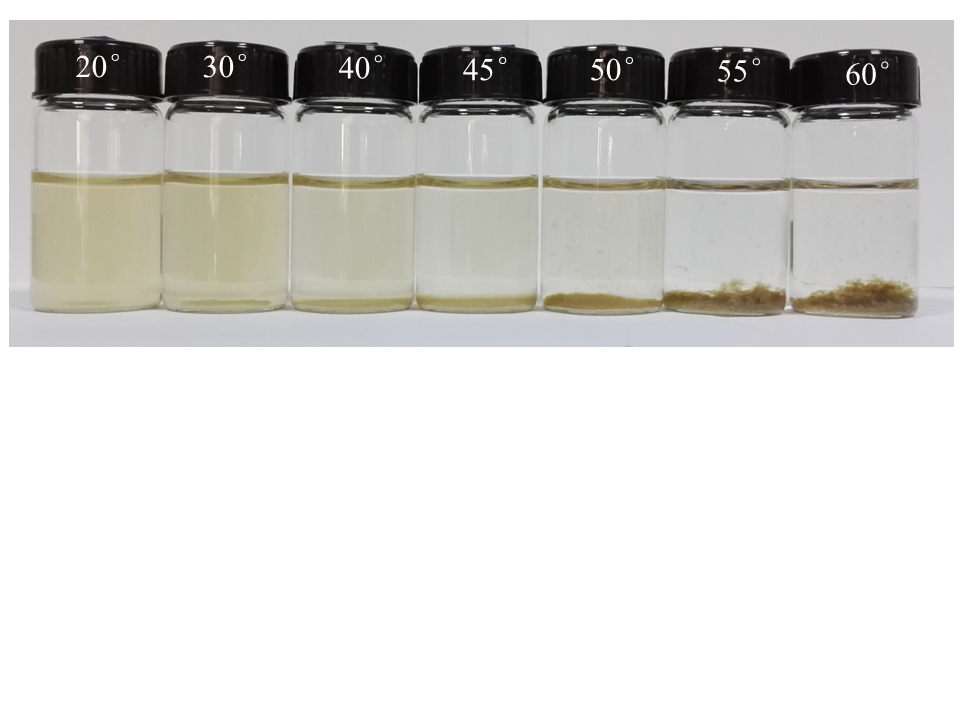


Figure S3. Photograph illustrating the inﬂuence of temperature on coagulation behaviors of GO after 24 h. C_(GO)initial_ = 25 mg/L, m/V = 0.1 g/L, pH = 5.0 ± 0.1.


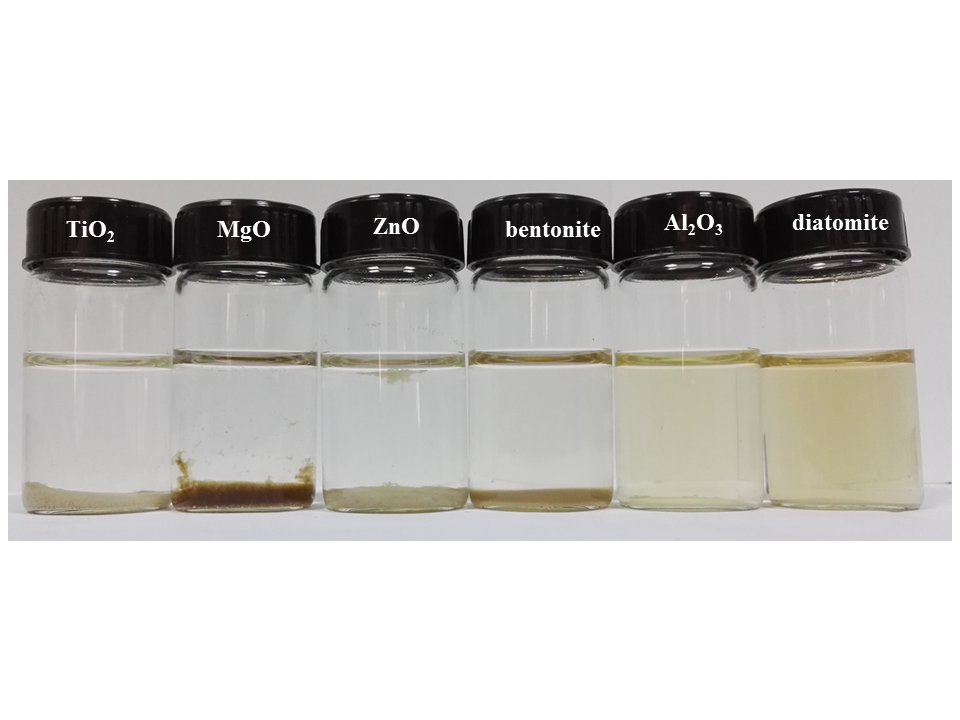


Figure S4. Photograph illustrating the coagulation behaviors of GO on different coagulants after 24 h. C_(GO)initial_ = 25 mg/L, m/V = 1 g/L, pH = 5.0 ± 0.1, T = 25 °C.


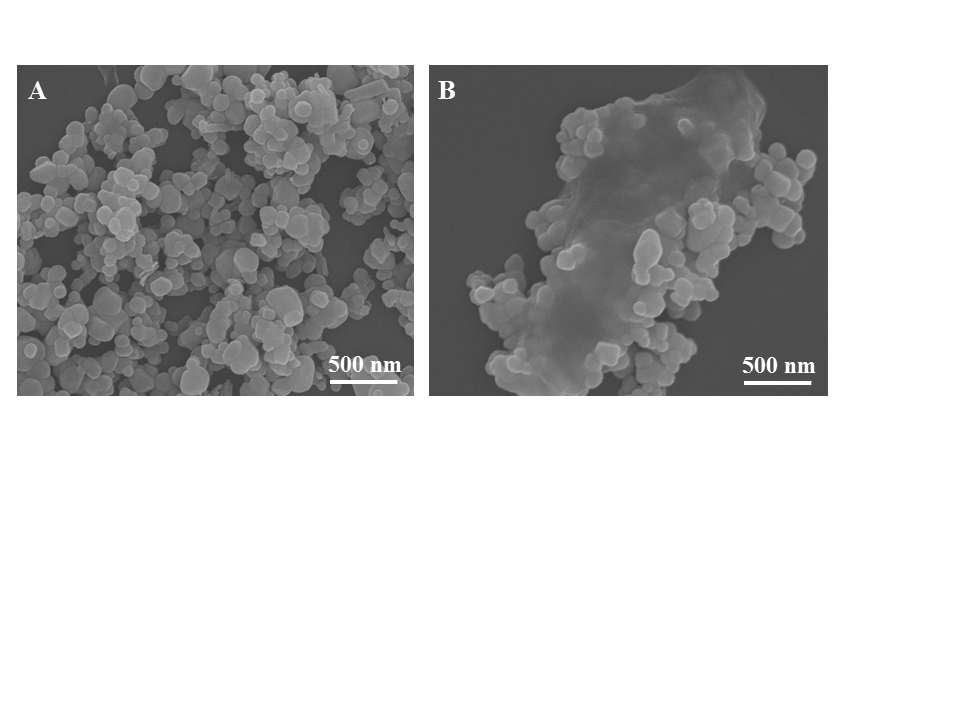


Figure S5. The SEM images of TiO_2_ before (A) and after (B) coagulation of GO.


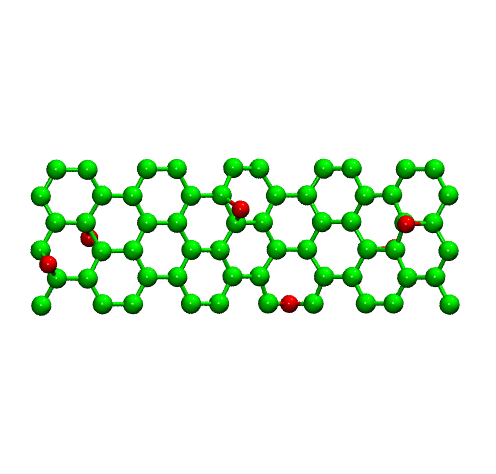


Figure S6. The optimized structure of GO.

Table S1. The calculated corresponding energies for different systems and the binding energy (*E*_b_) of GO+TiO_2_ system.

| *E*_GO_ | *E*_TiO2_ | *E*_GO+TiO2_ | *E*_b_(eV) |
| --- | --- | --- | --- |
| -576.79 | -1702.40 | -2284.98 | 5.79 |

References

1. Hummers, W. S. & Offeman, R. E. Preparation of graphitic oxide. *J. Am. Chem. Soc.* **80,** 1339-1339 (1958).

2. Perdew, J. P., Burke, K. & Ernzerhof, M. Generalized gradient approximation made simple. *Phy. Rev. Lett.* **77,** 3865-3868 (1996).

3. Methfessel, M. & Paxton, A. T. High-precision sampling for Brillouin-zone integration in metals. *Phys. Rev. B* **40,** 3616-3621 (1989).

4. Monkhorst, H. J. & Pack, J. D. Special points for Brillouin-zone integrations. *Phys. Rev. B* **13,** 5188-5192 (1976).
